# Suppression of top-down influence decreases neuronal excitability and contrast sensitivity in the V1 cortex of cat

**DOI:** 10.1038/s41598-021-95407-7

**Published:** 2021-08-06

**Authors:** Jian Ding, Xiangmei Hu, Fei Xu, Hao Yu, Zheng Ye, Shen Zhang, Huijun Pan, Deng Pan, Yanni Tu, Qiuyu Zhang, Qingyan Sun, Tianmiao Hua

**Affiliations:** College of Life Sciences, Anhui Normal University, Wuhu, 241000 Anhui China

**Keywords:** Neuroscience, Systems biology

## Abstract

How top-down influence affects neuronal activity and information encoding in the primary visual cortex (V1) remains elusive. This study examined changes of neuronal excitability and contrast sensitivity in cat V1 cortex after top-down influence of area 7 (A7) was modulated by transcranial direct current stimulation (tDCS). The neuronal excitability in V1 cortex was evaluated by visually evoked field potentials (VEPs), and contrast sensitivity (CS) was assessed by the inverse of threshold contrast of neurons in response to visual stimuli at different performance accuracy. We found that the amplitude of VEPs in V1 cortex lowered after top-down influence suppression with cathode-tDCS in A7, whereas VEPs in V1 did not change after sham-tDCS in A7 and nonvisual cortical area 5 (A5) or cathode-tDCS in A5 and lesioned A7. Moreover, the mean CS of V1 neurons decreased after cathode-tDCS but not sham-tDCS in A7, which could recover after tDCS effect vanished. Comparisons of neuronal contrast-response functions showed that cathode-tDCS increased the stimulus contrast required to generate the half-maximum response, with a weakly-correlated reduction in maximum response but not baseline response. Therefore, top-down influence of A7 enhanced neuronal excitability in V1 cortex and improved neuronal contrast sensitivity by both contrast gain and response gain.

## Introduction

The mechanisms that different brain regions mediate visual perception and perceptual learning remain poorly understood^1,2^. It is previously thought that visual perception depends primarily on feedforward signaling along the visual pathway from early processing stages to higher visual cortical areas^3^. In recent decades, an increasing body of evidence, especially studies on visual attention, indicate that top-down influence of higher-order brain areas on the primary visual cortex (V1) plays a critical role in the gating of visual perceptual activities^1,4,5^. However, the mechanisms of top-down influence on visual perception and information processing in the V1 and lower cortical areas remain elusive. Several electrophysiological studies indicate that the responses of neurons in the V1 cortex are modified after neuronal activity in the high-order cortex is inactivated by pharmacological administration^6^, cortical cooling^7,8^ and optogenetic manipulation^9^. Nevertheless, the results they report are diverse or even contradictory^6–8^. Therefore, the role and mechanism of top-down influence from higher cortical regions on neuronal activity in the low-level visual cortex need further exploration.

Several lines of evidences indicate that the cortical area 7 (A7) is a higher-order visual cortex of cats. Neuronal tracing studies show that A7 has direct corticocortical connections to the area 17 (V1), and receives input from a broad visual cortical areas, such as area 19, 20a, 20b, 21a and 21b^10,11^. Further, a recent study report that inactivation of A7 with local GABA application or nitrogen freezing significantly decreases the response amplitude of orientation maps in the V1 cortex^12^. These evidences indicate that A7 has an evident top-down influence on the neuronal activity in V1 cortex. Nevertheless, how top-down feedback of A7 affects neuronal excitability and contrast sensitivity in the V1 cortex remains unclear.

Visual contrast detection is a fundamental visual ability of human and animals in identifying object form, size and motion characteristics^13^. However, the underlying brain mechanisms remain unclear. Numerous previous studies focus on the role of V1 cortex in the visual contrast detection because of the similarity between contrast-response function of V1 neurons and the psychological contrast detection performance^14–16^. Further, the psychophysical contrast sensitivity versus stimulus spatial frequency (CSF) functions measured in cat is highly correlated with the neuronal CSF constructed from the response of V1 neurons^17^. In addition, practicing contrast detection can improve both psychological visual contrast sensitivity and contrast sensitivity of V1 neurons^18^. These studies suggest that neurons in the V1 cortex may play a critical role in the encoding of visual stimulus contrast. Nevertheless, it is unclear how higher-order visual cortical areas affect stimulus contrast encoding in the V1 cortex, although a few of studies have shown that attention can impact contrast detection threshold and neuronal contrast response functions in the higher-level visual areas^4,19^.

To explore the issues mentioned above, this study first examined changes of neuronal excitability in the V1 cortex by recording visually evoked field potentials (VEPs), a method that can measure membrane potentials of a large population of neurons, in the V1 area before and after top-down influence of A7 was modulated by a non-invasive tool of transcranial direct current stimulation (tDCS). This tDCS technique can reversibly affect neuronal activities in the stimulated local brain region^20,21^. In addition, the responses of V1 neurons to preferred visual stimuli with different contrast (0, 0.025, 0.05, 0.1, 0.2, 0.3, 0.4, 0.5, 0.6, 0.8 and 1.0) were recorded using in vivo single-unit recording techniques^17,18^ before and after tDCS in A7. The threshold of stimulus contrast (TC) that V1 neurons could respond to visual stimuli was computed by receiver operating characteristics (ROC) analysis^22^. The contrast-response function of each studied neuron was fitted with hyperbolic ratio function^17,18^ to acquire best-fitting parameters. By comparing changes of post- versus pre-tDCS parameters, we attempt to uncover the mechanisms of top-down influence on contrast encoding in the V1 cortex.

## Results

### Top-down influence of A7 on neuronal excitability in the V1 cortex

**Figure 1:**
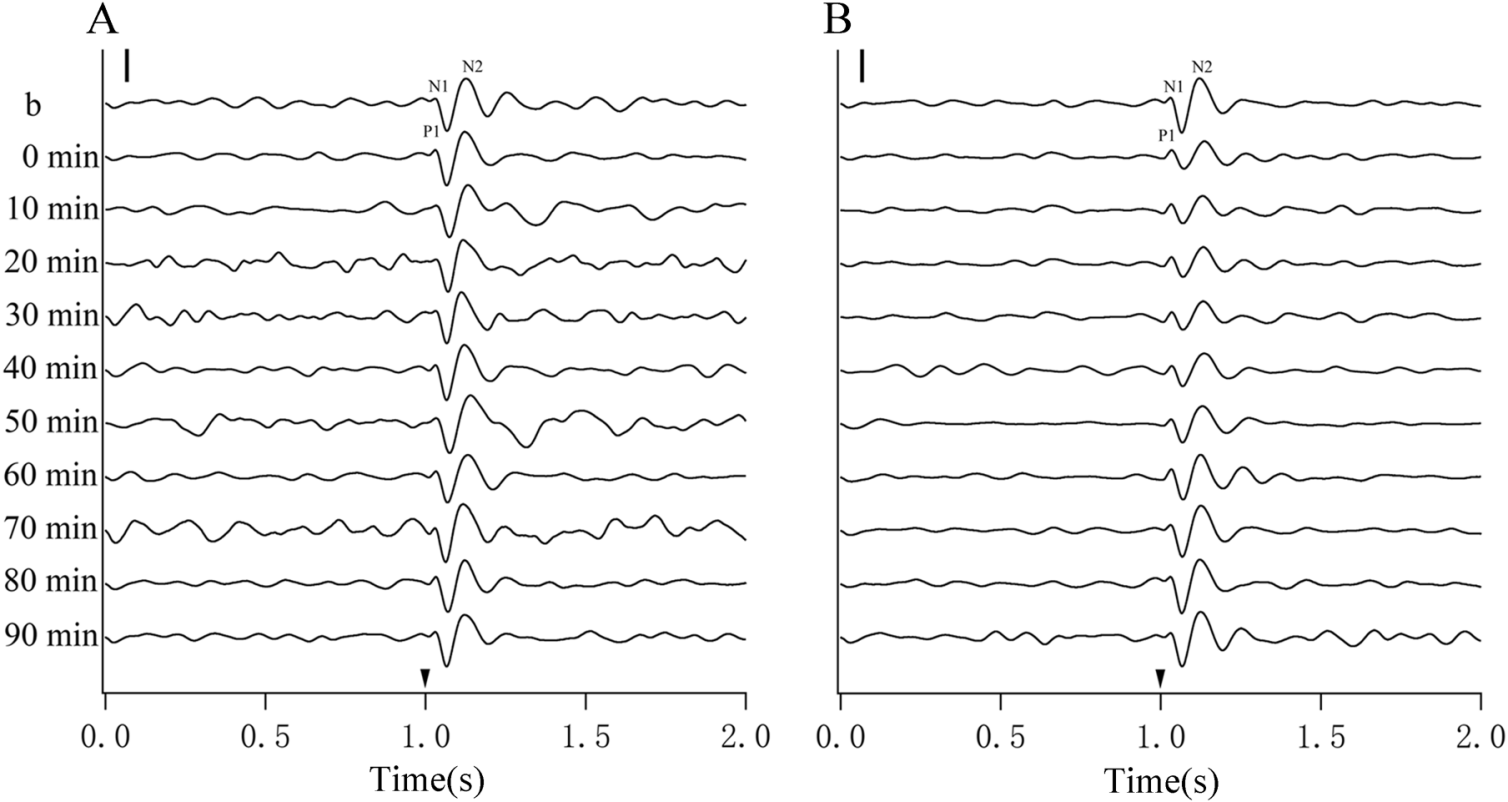
Samples of VEP voltage trace showing the three main components (wave N1, P1 and N2) and the changes of VEPs in the V1 cortex before (b) and at different time point (0, 10, 20, 30, 40, 50, 60, 70, 80 and 90 min) after the end of s-tDCS (**A**) and c-tDCS (**B**) in the area 7, respectively. Solid arrowheads indicate the onset of visual stimulation; The solid vertical scale bars in the upper left corner in A and B represent 100 μv. Image created using Igor (version 6.3.1.2, www.wavemetrics.com).

To examine how top-down influence of A7 affected neuronal excitability in the V1 cortex, we recorded VEPs in the V1 cortex before tDCS and at different time (0–90 min) after the end of tDCS in A7. The components of VEPs recorded in the V1 cortex, including an initial negative wave N1, a positive wave P1 and a late negative wave N2, were clearly identified both before and after sham (s)- and cathode (c)-tDCS in A7 (Fig. 1A,B), which were similar to observations in previous studies^23,24^, but showed lower amplitude in N1 wave than in P1 and N2 waves. The latency of wave N1, P1 and N2 of VEPs in the V1 cortex had a peak respectively at 21.7–39.2 ms, 46.8–76.4 ms and 100.1–141.1 ms after the stimulus onset, which were shorter than that measured in the area 21a of higher-order visual cortex^24^. The amplitude of VEPs was measured with peak-to-peak amplitude of N1P1 and P1N2 as previously described^23,24^.

**Figure 2:**
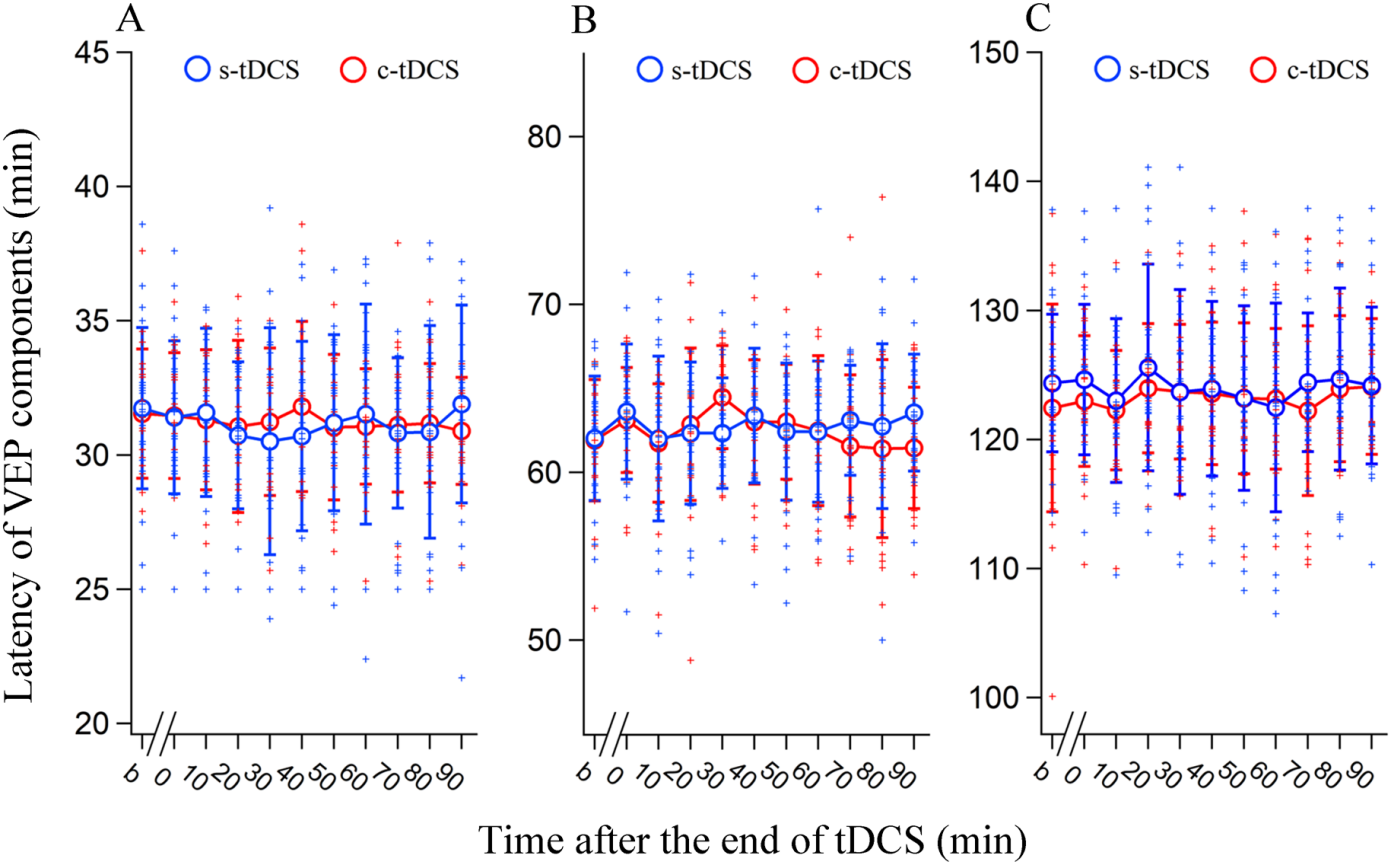
The latency at the peak of wave N1 (**A**), P1 (**B**), and N2 (**C**) of VEPs recorded in the V1 cortex across all cats before and at different time (0, 10, 20, 30, 40, 50, 60, 70, 80, and 90 min) after the end of tDCS in the area 7 (A7). The b indicates the measurement of VEP latency before tDCS. The blue and red open circles with error bars (SDs) indicate the mean latency measured at each time point before and after sham (s-) tDCS and cathodal (c-) tDCS in A7, respectively. The blue and red crosses represent individual data points of 24 measurements in four cats (six repeated measurements/cat) before and after s- and c-tDCS in A7, respectively. Image created using Igor (version 6.3.1.2, www.wavemetrics.com).

The mean latency of wave N1, P1 and N2 in the V1 cortex remained stable before and at different time point (0, 10, 20, 30, 40, 50, 60, 70, 80 and 90 min) after the end of tDCS in A7 (Fig. 2A–C). Statistical analyses showed that the mean latency of wave N1, P1 and N2 measured before and at different time point after the end of s- and c-tDCS in A7 had no significant difference (main effect of measurement time point: N1: F(10,506) = 0.591, p = 0.822; P1: F(10,506) = 0.855, p = 0.356; N2: F(10,506) = 0.539, p = 0.863); there was no interaction between measurement time point and tDCS: N1: F(10,506) = 0.860, p = 0.571; P1: F(10,506) = 0.894, p = 0.539; N2: F(10,506) = 0.360, p = 0.963). These results indicated that tDCS in A7 had no significant influence on the latency of VEPs in the V1 cortex.

**Figure 3:**
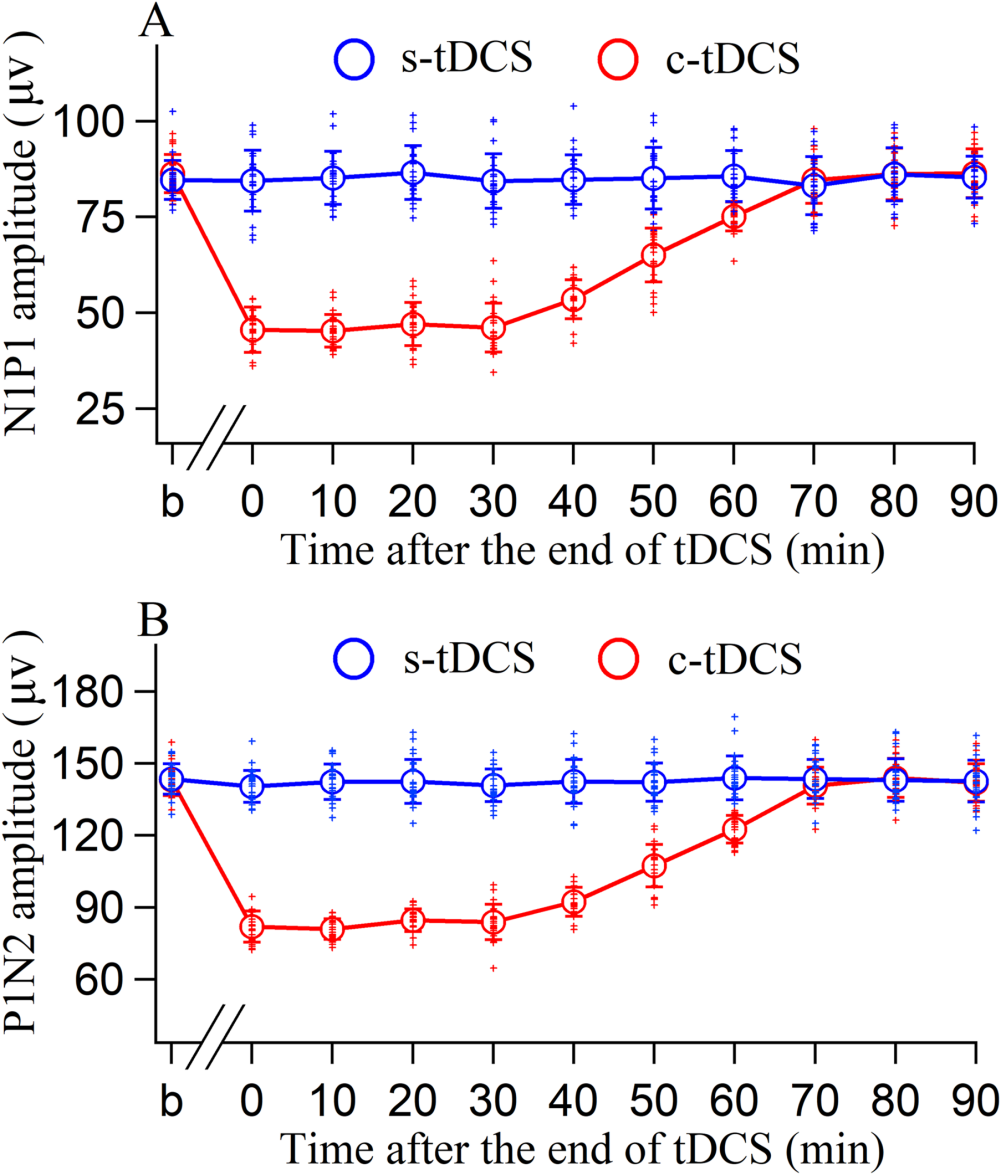
The peak-to-peak amplitude of N1P1 (**A**) and P1N2 (**B**) of the VEPs recorded in the V1 cortex across all cats before (b) and at different time point (0, 10, 20, 30, 40, 50, 60, 70, 80, and 90 min) after the end of tDCS in the area 7 (A7). The blue and red open circles indicate the mean amplitude (means ± SDs) measured at each time point before and after sham (s-) tDCS and cathodal (c-) tDCS in A7, respectively. The blue and red crosses represent individual data points of 24 measurements in four cats (6 repeated measurements/cat) before and after s- and c-tDCS in A7, respectively. Image created using Igor (version 6.3.1.2, www.wavemetrics.com).

The peak-to-peak amplitude of VEPs in the V1 cortex was also compared before and at different time points after tDCS. Two-way ANOVA showed that the mean amplitude of N1P1 and P1N2 measured before and at different time point after tDCS in A7 exhibited a significant variation [N1P1: F(10,506) = 102.928, p < 0.0001; P1N2: F(10,506) = 153.869, p < 0.0001], but the effect was dependent on tDCS polarity [N1P1: F(10,506) = 101.704, p < 0.0001; P1N2: F(10,506) = 172.037, p < 0.0001] (Fig. 3A,B). Therefore, we further compared VEP amplitude value at different measurement time point under s- and c-tDCS in A7, respectively. One-way ANOVA displayed that the mean N1P1 and P1N2 measured in the V1 cortex at different time point before and after s-tDCS in A7 had no significant difference [N1P1: F(10,253) = 0.444, p = 0.924; P1N2: F(10,253) = 0.431, p = 0.931]. Nevertheless, the mean N1P1 and P1N2 measured at different time point before and after c-tDCS in A7 exhibited significant difference [N1P1: F(10,253) = 248.202, p < 0.0001; P1N2: F(10,253) = 390.693, p < 0.0001]. Further post-hoc test indicated that the mean N1P1 measured at 0, 10, 20, 30, 40, 50 and 60 min after the end of c-tDCS was significantly decreased compared with that before c-tDCS (all p < 0.0001), whereas the mean N1P1 measured at 70, 80 and 90 min after c-tDCS did not differ significantly from that before c-tDCS (p = 0.314, 0.976, and 0.947). Similarly, the mean P1N2 value measured at 0, 10, 20, 30, 40, 50, and 60 min after the end of c-tDCS was also reduced compared with that before c-tDCS (all p < 0.0001), whereas the mean P1N2 measured at 70, 80, and 90 min after the end of c-tDCS showed no significant difference from that before c-tDCS (p = 0.191, 0.761, and 0.510) (Fig. 3A,B). These comparisons indicated that c-tDCS, but not s-tDCS, in A7 suppressed neuronal excitability in the V1 cortex as indicated by a significantly lowered VEPs amplitude (lowered up to 47.1% in N1P1 amplitude and 42.8% in P1N2 amplitude), and the effects lasted for 60–70 min.

**Figure 4:**
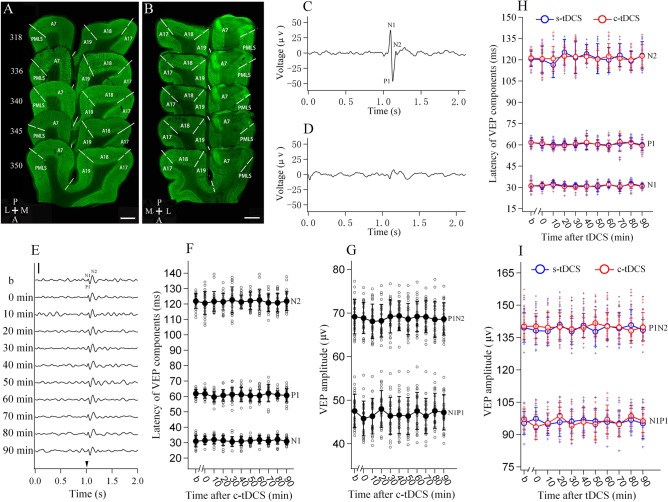
Showing effects of c-tDCS in the lesioned area 7 (A7) as well as c- and s-tDCS in the nonvisual cortex of area 5 (A5) on VEPs in the V1 cortex (area 17). (**A**–**G**) Effects of c-tDCS in A7 on VEPs in the V1 cortex after inactivation of neuronal activity in A7 by electrolytic lesions. A&B are samples of section images showing the morphology of normal A7 (**A**) and A7 with electrolytic lesions (**B**). The number on the left of each section indicates section number counted along the posterior-to-anterior (A–P) direction. The broken lines between A17, A18, A19, A7 and PMLS show estimates of the border between these area. The scale bar equals to 1000 μm. C&D show voltage traces of wave N1, P1 and N2 components of VEPs measured in A7 before and after electrolytic lesions, respectively. The pre-stimulus time is 1.0 s. (E) VEP voltage trace samples measured before (b) and at different time (0–90 min) after the end of c-tDCS in the lesioned A7. The solid vertical bar equals to 100 μv, and the arrowhead indicate the onset of visual stimulation. F&G show respectively the latency of wave N1, P1 and N2 as well the peak-to-peak amplitude of N1P1 and P1N2 of VEPs in the V1 cortex measured before (b) and at different time (0–90 min) after c-tDCS in the lesioned A7. The solid circles with error bars (mean ± SDs) indicate average value across four cats. The small open circles represent individual data point of 24 repeated measurements (6 repeated measures/cat). H&I show respectively the latency of wave N1, P1 and N2 as well the amplitude of N1P1 and P1N2 of VEPs in the V1 cortex measured before (b) and at 0–90 min after s- and c-tDCS in A5. The open color circles indicate average value across two cats. The color crosses represent individual data point of 12 repeated measurements. Images created using FV10-ASW (version 03.01.02.02, Olympus Corporation, www.olympus-sis.com), Igor (version 6.3.1.2, www.wavemetrics.com).

To confirm whether the lowered VEPs amplitude in the V1 cortex after c-tDCS in A7 was caused by alterations in top-down influence of A7 or by direct impact of current diffusion across cortical regions, we further examined c-tDCS related VEPs change in the V1 cortex after neuronal activity in A7 was abolished by electrolytic lesions. Morphological studies showed that almost all area of A7 in the left hemisphere was damaged by electrolytic lesions compared with that of normal A7 in the right hemisphere (Fig. 4A,B). VEPs recording also displayed that the main components of VEPs in A7 disappeared after electrolytic lesions compared with that before lesion (Fig. 4C,D). Statistical comparisons showed that the mean latency of wave N1, P1 and N2 of VEPs recorded in the V1 cortex before and at different time point after the end of c-tDCS in A7 exhibited no significant difference [N1: F(10,253) = 0.757, p = 0.67; P1: F(10,253) = 0.784, p = 0.644; N2: F(10,253) = 0.308, p = 0.979] (Fig. 4E,F). Furthermore, the mean value of peak-to-peak amplitude of VEPs measured in the V1 cortex at different time point after c-tDCS in A7 had no significant difference from that before c-tDCS [N1P1: F(10,253) = 0.708, p = 0.717; P1N2: F(10,253) = 0.316, p = 0.97] (Fig. 4E,G) although the VEPs amplitude of N1P1 and P1N2 in the V1 cortex after lesion of A7 was evidently lower than before lesion of A7 (Figs. 3A,B, 4G).

We also performed another experiment in two cats to examine VEP changes after tDCS application in a nonvisual cortex of area 5 (A5) adjacent to A7. Our results showed that the mean latency of VEPs in the V1 cortex recorded before and at different time point after s-tDCS and c-tDCS in A5 had no significant difference (N1: F(10,242) = 1.563, p = 0.118; P1: F(10,242) = 1.246, p = 0.262; N2: F(10,242) = 0.91, p = 0.525), there was no interaction between measurement time point and tDCS (N1: F(10,242) = 0.236, p = 0.992; P1: F(10,242) = 0.197, p = 0.996; N2: F(10,242) = 0.386, p = 0.952) (Fig. 4H). Further, the mean N1P1 and P1N2 amplitude of VEPs in the V1 cortex measured before and at different time point after both s- and c-tDCS in A5 exhibited no significant difference (N1P1: F(10,242) = 0.591, p = 0.82; P1N2: F(10,242) = 0.247, p = 0.991), with no interaction with tDCS condition (N1P1: F(10,242) = 0.711, p = 0.713; P1N2: F(10,242) = 0.473, p = 0.906) (Fig. 4I).

All analyses outlined above demonstrated that c-tDCS, but not s-tDCS, in A7 suppressed neuronal excitability in the V1 cortex. The observed effect in the V1 cortex lasted for about 60–70 min and recovered during 60–70 min after the end of c-tDCS. By contrast, s- and c-tDCS in the nonvisual cortex of A5 as well as c-tDCS in A7 after silence with lesion had no influence on VEPs in the V1 cortex, which strongly suggested that the c-tDCS effect of A7 on VEPs in the V1 should result from top-down influence of A7, not from direct current impact across cortical regions.

### Top-down influence of A7 on the contrast sensitivity of V1 neurons

To explore top-down influence of A7 on the contrast sensitivity (CS) of V1 neurons, we recorded the response of V1 neurons to visual stimuli with gradient luminance contrasts before and after s- or c-tDCS in A7, respectively. A total of 87 neurons with different preferred SFs (0.1–0.6 cycle/°) were included in data analysis. The contrast sensitivity (CS) of neurons with different preferred spatial frequencies (SFs) was evaluated by the inverse of threshold contrast (TC) at two performance criteria (70.7% and 79.3%) both before and after s- and c-tDCS in A7 (Table 1). We also examined the contrast sensitivity of some neurons by recording their response to stimuli with gradient contrasts after recovery from c-tDCS effect at the time point of 90 min after the end of c-tDCS^24–26^ (Table 1). A part of neurons lost hold of recording before the time point of recovery. The post- versus pre-tDCS CS value of V1 neurons with different preferred SFs were compared at two performance criteria, respectively.

**Table 1:** The cell number (CN) and the mean contrast sensitivity (CS) value of V1 neurons with different preferred spatial frequencies (SFs) under two performance criteria (70.7% and 79.3%) before and after sham (s)- and cathode c-tDCS in the area 7 as well as some neurons before c-tDCS and after recovery from c-tDCS effect.

d′	SFs	Before and after s-tDCS			Before and after c-tDCS			Before and after recovery		
		CN	CS pre	CS post	CN	CS pre	CS post	CN	CS pre	CS post
d1′	0.1	13	11.3 ± 2.2	11.0 ± 2.5	15	11.1 ± 2.8	6.6 ± 2.8	10	12.1 ± 2.6	10.7 ± 2.3
	0.2	15	16.1 ± 5.8	14.6 ± 4.4	14	15.0 ± 2.8	9.5 ± 3.0	8	15.0 ± 1.7	13.5 ± 2.6
	0.4	10	8.9 ± 3.2	10.9 ± 7.6	9	10.4 ± 2.8	5.8 ± 1.3	6	9.2 ± 2.5	8.9 ± 1.3
	0.6	6	4.7 ± 0.9	5.5 ± 1.5	5	8.2 ± 2.4	4.5 ± 1.0	3	8.1 ± 3.0	8.2 ± 1.2
d2′	0.1	13	7.9 ± 1.4	8.8 ± 2.5	15	7.1 ± 1.5	4.4 ± 1.5	10	7.5 ± 1.2	6.8 ± 1.8
	0.2	15	11.2 ± 4.5	11.9 ± 4.9	14	10.1 ± 2.4	6.3 ± 1.9	8	10.7 ± 2.4	9.4 ± 2.7
	0.4	10	6.3 ± 1.7	6.3 ± 1.6	9	7.9 ± 2.3	4.1 ± 0.8	6	6.9 ± 2.1	7.2 ± 1.4
	0.6	6	3.7 ± 0.8	3.5 ± 0.9	5	6.1 ± 2.1	3.4 ± 0.7	3	6.4 ± 2.5	6.0 ± 1.1

The CS value is expressed as mean ± SD. SFs is the preferred spatial frequencies (cycle/°) of V1 neurons. Pre and post indicate before and after tDCS, respectively.

**Figure 5:**
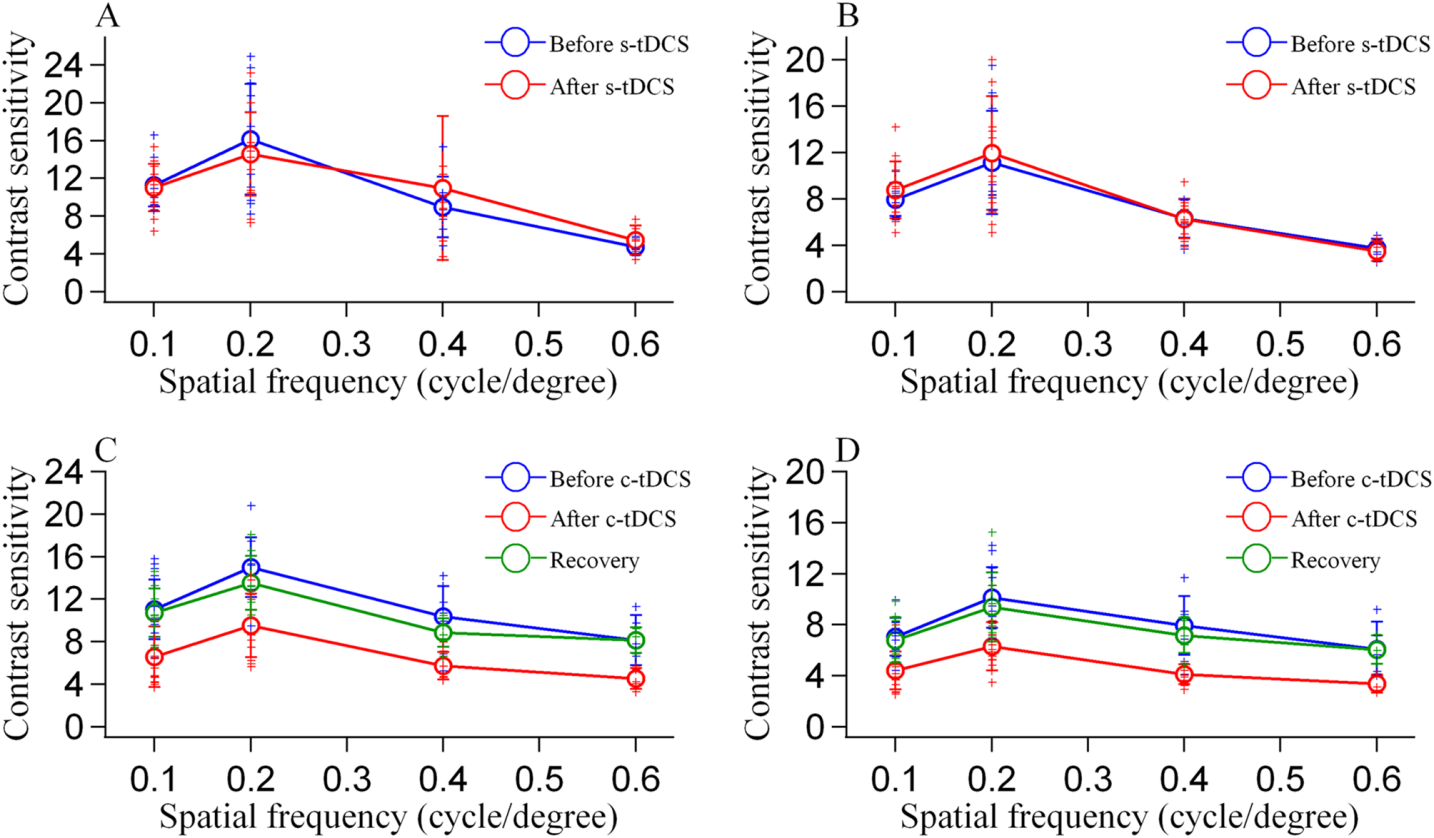
Contrast sensitivity of V1 neurons with different preferred spatial frequencies before and after sham (s)-tDCS and cathode (c)-tDCS in A7. The contrast sensitivity is expressed as the inverse of threshold contrast computed by ROC analysis at the performance accuracy of 70.7% and 79.3%, respectively. (**A**, **B**) contrast sensitivity of V1 neurons at performance accuracy of 70.7% (**A**) and 79.3% (**B**) before and after s-tDCS. (C&D) contrast sensitivity of V1 neurons at performance accuracy of 70.7% (**C**) and 79.3% (**D**) before and after c-tDCS in A7 as well as after recovery of c-tDCS effect. Crosses with different color in each plot represent individual data point from different V1 neurons. Image created using Igor (version 6.3.1.2, www.wavemetrics.com).

Two-way ANOVA showed that the mean CS of V1 neurons after s-tDCS in A7 had no significant difference from that before s-tDCS [main effect of tDCS: F(1,160) = 0.217, p = 0.642]; the effect exhibited no interaction with SF [F(3,160) = 0.265, p = 0.85] and performance criterion [F(1,160) = 0.008, p = 0.929] although the mean CS had a significant variation between different SFs [F(3,160) = 37.889, p < 0.0001] and between different performance criteria [F(1,160) = 23.423, p < 0.0001] (Fig. 5A,B).

Unlike s-tDCS, c-tDCS in A7 exerted an evident influence on CS of V1 neurons. Two-way ANOVA showed that the mean CS of V1 neurons after c-tDCS in A7 was significantly decreased compared with that before c-tDCS [main effect of tDCS: F(1,156) = 105.564, p < 0.0001]; this effect was independent of SF [F(3,156) = 0.799, p = 0.496] and performance criterion [F(1,156) = 2.968, p = 0.087] (Fig. 5C,D).

We also compared the CS of some neurons (27/43) after recovery from c-tDCS effect with that before c-tDCS in A7. Two-way ANOVA showed that the mean CS of V1 neurons after recovery of c-tDCS effect displayed no significant difference from that before c-tDCS [main effect of tDCS: F(1,92) = 2.176, p = 0.144]; there was no significant interaction between c-tDCS effect and SF [F(3,92) = 0.606, p = 0.613] as well as between c-tDCS and performance criterion [F(1,92) = 0.084, p = 0.773].

The comparisons displayed above indicated that the contrast sensitivity of V1 neurons decreased significantly after suppressing top-down influence of A7 with c-tDCS, and could recover after the tDCS-induced top-down influence vanished. By contrast, s-tDCS in A7 had no impact on neuronal contrast sensitivity in the V1 cortex.

### Mechanisms of top-down influence of A7

To explore the mechanisms underlying top-down influence of A7 on the contrast sensitivity of V1 neurons, we fitted the contrast-response function of each neuron with the hyperbolic ratio equation [Eq. (1)]^17,18^ before and after c-tDCS in A7 as well as after recovery of c-tDCS effect (S-Fig. 1). Based on the contrast-response function, contrast sensitivity changes of V1 neurons could be caused by following factors^18,27,28^: (1) contrast gain (C_50_) due to a parallel shift of contrast-response function along x-axis of stimulus contrast; (2) change in the exponents of contrast response function (N); (3) change in the spontaneous activity (M); (4) response gain due to alteration of maximum visually evoked response (R_max_). Therefore, we systematically compared the best-fitting parameters of the hyperbolic ratio function [Eq. (1)] before and after top-down influence of A7 was modulated by c-tDCS. Because the top-down influence of A7 on contrast sensitivity of V1 neurons was independent of neuronal preferred SFs, the comparison of parameter C_50_, N, M and R_max_ was pooled across all studied neurons with different SFs.

**Figure 6:**
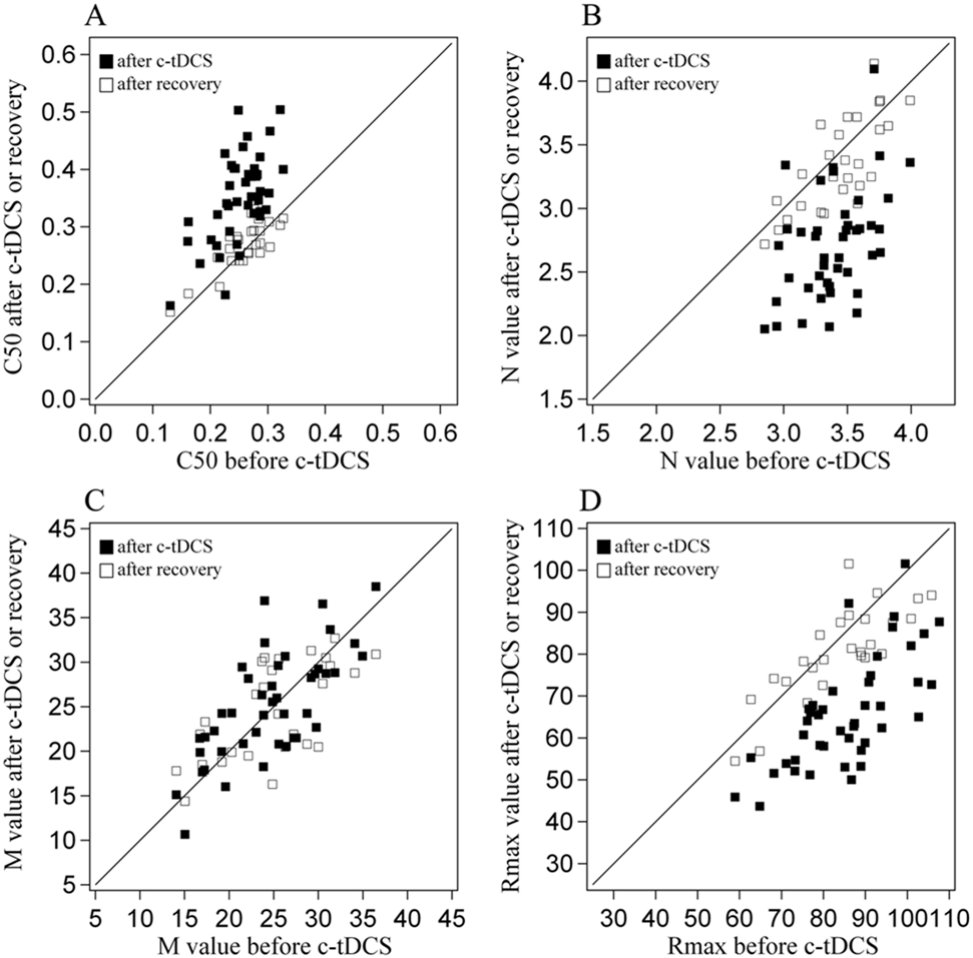
Scatter plots showing the best-fitting parameter value of contrast-response functions for V1 neurons across all preferred spatial frequencies (SFs). Solid squares in each plot represent individual data after versus before cathode-tDCS, and open squares indicate data of post-recovery versus before cathode-tDCS. (**A**) Stimulus contrast generating the half amplitude of maximum visually evoked response (C_50_); (**B**) Exponents of contrast response function (N); (**C**) Spontaneous activity or baseline response (M); (**D**) Maximum visually evoked response (Rmax). Image created using Igor (version 6.3.1.2, www.wavemetrics.com).

The C_50_ value of most V1 neurons increased after c-tDCS relative to before c-tDCS in A7 (Fig. 6A). Two-way ANOVA showed that the mean C_50_ of V1 neurons after c-tDCS in A7 was significantly higher than that before c-tDCS (mean increase of 39.4%) [main effect of tDCS: F(1,78) = 47.804, p < 0.0001], and this effect was independent of neuronal preferred SF [F(3,78) = 0.078, p = 0.972]. Conversely, the N (exponents) value of most V1 neurons reduced after c-tDCS relative to before c-tDCS in A7 (mean reduction of 20.8%) (Fig. 6B). ANOVA comparison exhibited that the mean N value of all neurons after c-tDCS in A7 was significantly decreased compared with that before c-tCS [F(1,78) = 58.324, p < 0.0001]; the effect had no interaction with neuronal preferred SF [F(3,78) = 0.176, p = 0.912]. The M value of most neurons after c-tDCS was equal or close to that before c-tDCS (Fig. 6C). ANOVA analysis displayed that the mean M value of all neurons after c-tDCS had no significant variation from that before c-tDCS [main effect of tDCS: F(1,78) = 0.071, p = 0.791]; there was no interaction between tDCS effect and neuronal SF [F(3,78) = 0.087, p = 0.967]. However, the R_max_ value of most neurons was reduced (mean reduction of 22.6%) after c-tDCS relative to before c-tDCS in A7. ANOVA analysis exhibited that the mean R_max_ of all neurons after c-tDCS was significantly lowered when compared with that before c-tDCS [F(1,78) = 48.809, p < 0.0001]; this effect was independent of neuronal SF [F(3,78) = 0.481, p = 0.697].

We also compared the mean C_50_, N, M and R_max_ of some neurons at 90 min after the end of c-tDCS (vanishing of tDCS effect) with that before c-tDCS in A7. The results showed that the C_50_, N, M and R_max_ value of most studied neurons after vanishing of c-tDCS effect became nearly equal or close to that before c-tDCS in A7 (Fig. 6A–D). ANOVA comparisons showed that the mean C_50_, N, M and R_max_ of all recorded neurons after c-tDCS effect wearing off had no significant difference from that before c-tDCS in A7 [C_50_: F(1,46) = 0.187, p = 0.668; N: F(1,46) = 0.54, p = 0.466; M: F(1,46) = 0.172, p = 0.68; R_max_: F(1,46) = 0.975, p = 0.329]; the effect had no interaction with neuronal preferred SF [C_50_: F(3,46) = 0.057, p = 0.982; N: F(3,46) = 0.029, p = 0.993; M: F(3,46) = 0.185, p = 0.906; R_max_: F(3,46) = 0.004, p = 1.0].

All analyses above demonstrated that suppression of top-down influence with c-tDCS in A7 increased C_50_, but reduced N and R_max_ of the contrast-response function of V1 neurons. These effects could recover after the c-tDCS effect vanished. To explore whether the top-down effects on C_50_, N and R_max_ were mutually related or not, we calculated the correlation between changes in C_50_, N and R_max_. The results showed that no significant correlation was found between changes in C_50_ and N (r^2^ = 0.226, p = 0.146). There was a significant but low correlation between alterations of C_50_ and R_max_ (r^2^ = 0.361, p = 0.017) as well as between changes of R_max_ and N (r^2^ = 0.464, p = 0.002). This analyse indicated that top-down influence of A7 may affect contrast sensitivity of V1 neurons through multiple mechanisms of contrast gain and response gain.

## Discussion

### Top-down influence on neuronal activity in the V1 cortex

An increasing body of evidences have demonstrate that top-down influence plays a critical role in visual perceptual detection and learning^2,4^. The underlying mechanisms remain elusive^1,29^. Physiological investigations in recent years indicate that top-down influence from high-level visual cortex can modulate visual information encoding by affecting neuronal response in the primary visual cortex (V1) or low-level visual areas. Nevertheless, the results reported by different authors are diverse or even contradictory^8,29–32^. Numerous studies show that top-down input from high-level visual and even nonvisual cortex primarily facilitate neuronal response in the V1 area^6,7,9,12,33^. Conversely, other authors find that top-down influence exhibits an inhibitory effect on the response of V1 neurons^8,32^. Moreover, some studies even report a multiple top-down influence of both enhancement and suppression on neuronal response in the V1 cortex^30,31^. Reasons underlying these arguments remain unclear, but the following two factors may contribute. First of all, previous studies used different techniques to modulate neuronal activity of high-level cortical areas, which may cause variation of top-down effects in the time course, strength and reversibility. For example, application of GABA or GABA agonists in the high-level cortex may have difficulty in equally driving drug diffusion time course and range, and thus could lead to difference in top-down effects^6,32^. Cooling a cortical region may have a risk of impairing neuronal metabolism near the cooling site or causing incomplete recovery, and could also evoke differential top-down effects^7,8^. Optogenetic technique can precisely modulate neuronal activity in the local circuitry^9,33^, but has a limitation in controlling neuronal response at a large scale of a cortical region. Manipulation of top-down influence by visual attention is noninvasive and reversible, but subject to fluctuation with task demand and brain state^5^. On the other hand, the reported conflicts of top-down influence could also arise from the difference in the measurement of neuronal activities in the primary or low-level visual cortex. For instance, single- or multi-unit recording can measure neuronal response with a high temporal resolution, but at a trade-off with low spatial resolution in cell sampling^7,9,31–33^. Measurement with neural imaging techniques acquires signals of a large population of neurons, but has limitation in temporal resolution^6,12^.

The current study examined the top-down effects of the area 7 (A7), a high-level visual cortex of cat^10–12^, on neuronal activity in the V1 cortex (area 17). The top-down influence of A7 was modulated using tDCS, a noninvasive tool that could reversibly regulate neuronal activity at stimulated local brain region with a controllable time course^20,21,24^. Neuronal activity in the V1 cortex was assessed using multiple techniques, including recording of visually evoked field potentials (VEPs) that could measure the membrane potentials of a large population of neurons^34^ as well as extracellular single-unit recording with high temporal resolution. Our results showed that c-tDCS, but not s-tDCS, in A7 significantly decreased not only VEPs amplitude but also the maximum visually evoked responses (R_max_) of single-units in the V1 cortex. The observed changes of VEPs and single-unit response in the V1 after c-tDCS in A7 was not due to a variation of anesthesia level before and after c-tDCS because administration of anesthetic-urethane (20 mg/kg body weight/h) was kept constant, and the animal heart rate and ECG were maintained in the same normal range throughout the eletrophysiological recording. Further, the observed suppression of V1 neuronal activity after c-tDCS in A7 was not caused by a direct current influence on the V1 cortex because: (1) c-tDCS in A7 with neuronal activity abolished by electrolytic lesions had no significant impact on VEPs in the V1 cortex; (2) s- and c-tDCS in the nonvisual cortical area 5 (A5) adjacent to A7 also exhibited no significant influence on V1 VEPs. Therefore, the neuronal activity reduction observed in the V1 should result from top-down influence suppression in A7. Specifically, the mean single-unit response in the V1 decreased by 22.6% after c-tDCS in A7, which was close to the previously reported firing rate reduction of V1 neurons (about 22%) in response to simple grating stimuli after top-down influence of area 21a was suppressed by cooling under anesthesia state^7,35^. Although top-down effects have been shown to be strongly reduced under anesthesia^36^, this study observed a large VEP amplitude changes (decreased by 47.1% in N1P1 and 42.8% in P1N2) in the V1 after top-down suppression. This could be because VEPs measures the membrane potentials from a large population of neurons^24,34^ and thus may be easily affected by tDCS-induced changes in synaptic input^24,26,37^. It is noteworthy that both the initial component (N1P1) and late component (P1N2) of VEPs in the V1 cortex were significantly affected by top-down suppression of A7. This is related to a recent debate about whether top-down influence can modulate the initial response of feedforward input to the V1 cortex^38–41^. Some authors report top-down facilitation effect on the late-phase neuronal response^41^ or late ERP components (90–140 ms)^38^, whereas others find a strong attentional modulation on the initial response of VEP component (57 ms) and population response during the first wave of visual processing (< 100 ms) in the human V1 cortex^39,40^. It is likely that the top-down effect on the initial and late response components in the V1 may depend on the strength of top-down influence during different tasks^39^ or the source of top-down connections from different cortical areas to the V1 cortex^42,43^ or types of visual signals such as figure-ground stimuli^41^. Considering that A7 has a weblike and direct projections to the V1^10,12^, and tDCS manipulation may trigger a large scale of top-down influence from A7, it could be reasonable that visual stimulation with full-screen size gratings may potentiate a drastic top-down effect on VEPs, even the initial VEP components, in the V1 as observed in this study. Additional studies are needed to examine these possibilities.

In conclusion, our results suggest that suppression of top-down influence with c-tDCS in A7 decreases neuronal activity in the V1 cortex, which is consistent with some of previous studies using neural imaging measurement^6,12^ and some single-unit recording studies that report facilitatory top-down effects on most neurons in the V1 or low-level visual cortex^7,9,33,41^, and is also supported by neuronal tracing studies that identify most feedback neurons as excitatory cells^43,44^ although top-down modulation may mediated by a mechanism of disinhibition^41^. Our result is opposite to the results of other studies that report inhibitory top-down effects of V2/V3 cortex on V1 neurons in primate and mouse^8,29,32^. This suggests that the property of top-down influence may also vary with the source of feedback from different high-level cortical regions or depend on characteristic neuronal circuitry in different animal species. For instance, a previous study reports that feedback input from mouse frontal cortex can activate different types of inhibitory neurons in the local circuitry of V1 cortex^9^. This may explain why some authors observe a bidirectional top-down influence of enhancement and suppression on the response of V1 neurons^30,31^. Further studies are needed to clarify these conflicts.

### Top-down influence of A7 on contrast sensitivity of V1 neurons

Detection of luminance contrast is critical to the visual perception of object form, size and motion features^45^. The brain mechanisms is under debate. A considerable number of studies highlight the role of V1 cortex in the contrast detection because of the similarity between contrast-response function of V1 neurons and the psychological contrast detection performance^15^. Further, some studies show that psychophysical performance in signal contrast detection can be predicted from the pooled neuronal response to stimulus contrasts in the V1 or early visual cortical areas^14–16^. Additionally, previous study also show that the psychophysical contrast sensitivity versus stimulus spatial frequency (CSF) functions measured in cat is highly correlated with the neuronal CSF in the V1 cortex^17^, and perceptual learning of contrast detection can concurrently improve psychological contrast sensitivity and contrast sensitivity of V1 neurons around the trained spatial frequency^18^. These evidences suggest that neurons in the V1 cortex play a critical role in the encoding of visual stimulus contrast. However, other studies show that top-down influence driven by visual attention and cognition can enhance contrast detection in vision^4^, which raises the possibility that top-down influence may modulate contrast perception by increasing neuronal contrast sensitivity in the V1 or low-level visual areas. However, direct physiological evidence in favor of this inference is quite limited^9,19,29^ or remains uncertain^7,46^. On the other hand, the mechanisms of top-down influence on neuronal contrast sensitivity in the low-level visual cortex are not yet clear. Some authors suggest that attention-driven feedback causes a contrast gain as indicated by a parallel shift or proportional scaling of neuronal contrast response functions in the V4 or early cortical areas^19,27^. Others report a neuronal response gain at low and/or high contrast after activation of top-down influence^9,47^. Still others show that an increased input baseline in the neural responses may account for the attention-induced enhancement in perceived stimulus contrast^4^. Reasons leading to this variation are likely related to the instable control of attention^5,48^.

This study examined the changes in contrast sensitivity of V1 neurons before and after top-down influence of A7 was reversibly modulated using a noninvasive tool of tDCS. The contrast sensitivity of V1 neurons was assessed by the inverse of the threshold contrast that V1 neurons could detect visual stimuli at different performance accuracy using a precise measurement of Receiver Operating Characteristics (ROC) analysis. The results showed that the mean contrast sensitivity of V1 neurons varied with their preferred spatial frequencies (with a peak around 0.2 cycle/degree), consistent basically with previous observations^17,18^. However, the mean value of neuronal contrast sensitivity measured in this study, even at the peak spatial frequency (16.1), was greatly lower than that measured in behaving cats using a mean stimulus luminance of 16 cd/m^2^^49^ because the pupils of cats in the current study were dilated and produced poorer image quality than vision with natural pupils. Statistical comparisons showed that the contrast sensitivity of V1 neurons with different preferred SFs were significantly decreased at both low and high correct performance accuracy after c-tDCS when compared with that before c-tDCS in A7. This effect could recover after c-tDCS effect vanished. Therefore, our results indicate that top-down influence of A7 increases contrast sensitivity of V1 neurons, which is consistent with previous studies about effects of feedback from attention-related high brain regions^9,19^. Further comparisons of best-fitting parameters of neuronal contrast-response functions showed that top-down suppression after c-tDCS in A7 increased the stimulus contrast of V1 neurons in generating the half maximum visually-evoked response (C_50_) and reduced the maximum response (R_max_), but not the spontaneous activity. Specifically, there was a low and significant correlation between changes of C_50_ and R_max_ (r^2^ = 0.361, p = 0.017), but not between the change of C_50_ and exponent (N) of contrast-response function (r^2^ = 0.226, p = 0.146). This result suggests that top-down influence of A7 may affect contrast sensitivity of V1 neurons through a combined mechanism of contrast gain and response gain, which is consistent with the attentional effects on contrast discrimination^50^, but different from a single mechanism of response gain caused by surround suppression in V1 neurons^28^ and top-down effects on neuronal contrast sensitivity in the area V4 and MT of monkeys^47,51^ as well as attention-induced contrast gain of BOLD response in human visual cortex^27^. Therefore, the mechanism of top-down influence on neuronal contrast sensitivity in the visual cortex may vary with multiple factors, such as origin of higher-order brain regions, target cortical areas receiving top-down input and even species. Further studies are required to clarify these possibilities.

In summary, the current study indicated that top-down influence of A7 could increase neuronal excitability in the V1 cortex, and improve contrast sensitivity of V1 neurons by a combined mechanism of contrast gain and response gain.

## Material and methods

### Animals

Twelve young adult cats (age 1–3 years, body weight 2.8–3.9 kg) were used in this study. All subjects were healthy domestic cats provided by Nanjing Qing-Long-Shan Animal Breeding Farm (Jiangning District of Nanjing, Certificate No. SX1207). Before experiment, animals were reared in the laboratory under a room temperature of 25 °C for 3–5 days to accommodate new environment. All animals could get clean food and water freely. Each animal was fasted for 12 h before the experiment. All experiments in this study were performed strictly in accordance with the National Institutes of Health Guide for the Care and Use of Laboratory Animals, and conformed to the principles and regulations as described in the ARRIVE guidelines (Animal Research: Reporting of In Vivo Experiments). All animal treatments were approved by the Animal Welfare Ethics Committee of Anhui Normal University (approval NO. NS2017001).

### Examination of top-down influence on neuronal excitability in V1 cortex

To confirm whether and how top-down influence of A7 affects neuronal excitability in the V1 cortex, we recorded visually evoked field potentials (VEPs) in the V1 area of four cats before and after neuronal activity of A7 was suppressed by cathode (c-) tDCS. Sham (s-) tDCS in A7 was used as a control. To prove the specificity of top-down influence on VEPs change in the V1 cortex, we examined pre- and post-tDCS VEPs alterations in the V1 area after the neuronal activity of A7 was abolished by electrolytic lesion. To further confirm the top-down influence of A7, we also did another experiment in two cats and observed VEP changes in the V1 cortex before and after s- and c-tDCS in a nonvisual cortex of area 5 (A5).

### Recording preparations

The recording preparations were similar to that described in our previous studies^17,18,24^. The cat was first anesthetized with ketamine HCl (40 mg/kg, im) and xylazine (2 mg/kg, im). Intubation of tracheal and intravenous cannulae was performed under sterile preparation. After the cat was fixed in a stereotaxic apparatus, glucose (5%)-saline (0.9%) solution containing a mixture of urethane (20 mg/kg body weight/h) and gallamine triethiodide (10 mg/kg body weight/h) was infused intravenously to maintain necessary anesthesia and paralysis. Artificial respiration was performed, and expired pCO2 was kept at approximately 3.8%. Heart rate (approximately 180–220 pulses/min) and electrocardiogram were monitored throughout the experiment in order to assess the level of anesthesia and ensure the animals were not responding to pain. The body temperature (38 °C) was maintained using a heating blanket. Pupils were maximally dilated with atropine (0.5%), and artificial tears was applied to protect the cornea.

For application of tDCS, a three-dimensional printed plastic trough (rectangle shape with a section of 8 × 6 mm and a height of 10 mm) was implanted on the skull over A7 (Horsley–Clarke coordinates: A0–A8/L6–L12)^10,12^ or A5 (Horsley–Clarke coordinates: A11–A19/L6–L12) ^10,52–54^ (S-Fig. 2) of left hemisphere using dental cement. For recording of VEPs in the V1 cortex, a small hole (4 × 3 mm) was drilled on the skull over the central V1 area^7,10,12^ (Horsley–Clarke coordinates: P2–6/L2–4) (S-Fig. 2) of left hemisphere. A chloride (Ag/AgCl) silver wire electrode (extending from P2 to P6, with an impedance of 0.3–0.5 MΩ) was placed on the surface of the dura over V1 area. After filling with 4% agar, the small hole was sealed with tissue adhesive and fixed with dental cement.

### Application of transcranial direct current stimulation

Administration of c- and s-tDCS in A7 or A5 was performed with a HD-tDCS stimulator (Soterix Medical, USA). A metal pin-type electrode (cathode) was placed in the tDCS trough filled with 0.9% saline for conductance. The reference electrode (saline-soaked rubber electrode, 3 × 3 cm) was placed on the neck skin after the hair over the intended site was clipped and cleaned with alcohol swabs. The output current intensity was maintained at 1 mA. At the onset and offset of stimulation, current was slowly ramped up and ramped down over about 15 s to avoid sudden current change as described previously^55,56^. The s-tDCS was applied using the same procedure as c-tDCS except that the tDCS current was ramped down to zero after ramping up at the onset of stimulation, but ramped up and ramped down again at the end of sham stimulation^24,55,56^. The application of c- and s- tDCS was performed in an interleaved order and repeated 6 sessions in each cat. Because previous studies reported that tDCS-induced effects lasted for 60–90 min^24–26^, we set the interval between tDCS sessions at least 90 min. The duration of each tDCS session was maintained at 15 min.

### VEPs recording in the V1 cortex

The visually evoked field potentials (VEPs) in the V1 cortex were recorded with the embedded silver wire electrode. Signals were amplified with a microelectrode amplifier (Dagan 2400A, USA) (gain 1000, band-pass filtered between 1 and 200 Hz). The VEPs were recorded repeatedly before and at different time (0–90 min, with recording interval of 10 min) after the end of c- and s-tDCS in A7, respectively. VEPs collection at each time point consisted of 30 trials of visual stimulus presentation. Visual stimuli were horizontal sinusoidal gratings (full screen size with spatial frequency 0.2 cpd, temporal frequency 2 Hz and contrast 100%) generated by a PC computer with the aid of Matlab programs based on Psychotoolbox extensions. The stimuli had a fixed mean luminance of 19 cd/m^2^ and were presented for 0.5 s on a CRT (resolution 1024 × 768 pixels, refresh rate 75 Hz) positioned 57 cm from the animal’s eyes. Before the presentation of each trial of stimulus, baseline VEPs were recorded during 1 s period while the mean luminance was shown on the CRT. VEP signals were averaged across 30 rehearsals and filtered (60 Hz notch filter, 1–100 Hz bandpass) using igor software programs. VEPs were analysed in terms of peak latency of wave N1, P1 and N2 components as well as peak-to-peak amplitude of N1P1 and P1N2 as described previously^23,24,57^. Statistical difference between pre- and post-tDCS VEPs was determined with ANOVA and and ANOVA with post hoc pairwise tests.

### Electrolytic lesion in A7

To confirm whether changes of VEPs in the V1 cortex were resulted from top-down influence of A7 or from direct current influence of tDCS, we also examined tDCS-induced alterations of VEPs in the V1 area after neuronal activity in A7 was abolished by electrolytic lesion.

After the skull over A7 was opened carefully, the electrolytic lesion was performed with a stainless-steel electrode^58,59^ that penetrated vertically within a depth of 1200 μm from the pial surface at multiple locations in A7 (within a range at Horsley-Clarke coordinates: A0.5–7.5/L6.5–11.5, with a spacing of about 1–1.5 mm between different lesion locations). Lesions were made with anodal constant direct current at each location (1 mA for 15 s). To confirm if electrolytic lesions could abolish neuronal activity in A7, we recorded visually evoked field potentials (VEPs) on the surface of A7 using a glass-coated silver-wire electrode (with an impedance of ~ 0.5 MΩ) before and after lesions in A7. VEPs collection consisted of 30 trials of visual stimulus presentation both before and after lesions in A7. Visual stimuli were horizontal sinusoidal gratings (full screen size with spatial frequency 0.2 cpd, temporal frequency 2 Hz and contrast 100%) generated by a PC computer with the aid of Matlab programs. After completion of lesions, the exposed A7 was closed with the piece of repaired skull using tissue adhesive and dental cement, and the tDCS trough was re-implanted on the skull over A7.

### Histology examination

After VEPs recording, the cat’s brain tissue was harvested and used for histological study according to methods described previously^43^. Briefly, the cat was deeply anesthetized with ketamine HCl (80 mg/kg, im) and then transcardially perfused with 0.9% saline followed by 2% paraformaldehyde in 0.1 M phosphate buffered saline (PBS). The brain tissue on both hemispheres was removed and post-fixed overnight in 2% paraformaldehyde at 4 °C. On the next day, the cerebral cortex containing visual cortical area A17, A18, A19, A21a, PMLS and A7 was dissected and cryoprotected by sequential incubation in 10% (2 h), 20% (2 h) and 30% (overnight) sucrose until tissue sinking. Then, the brain tissue was embedded in OCT compound (Tissue-Tek, 4583, Sakura Finetek Inc., California, USA), and coronal sections were cut at a thickness of 40 µm using a Leica cryostat (Leica Biosystems Inc., Buffalo Grove, IL, USA). Serial frozen sections were collected in order, placed in wells filled with cryoprotectant solution (ethylene glycol-based; 30% ethylene glycol, 30% sucrose, 1% PVP-40, in 0.1 M Phosphate buffer pH 7.4) and temporarily stored at − 20 °C for subsequent immunoreactive labelling of cortical neurons. The free-floating sections were first incubated overnight at 4 °C with rabbit anti-NeuN (1:1000, ab177487, Abcam, Shanghai, China). After several washes in PBS, sections were then incubated with the secondary antibody (goat anti-Rabbit IgG H&L, Alexa Fluor 488, 1:1000, ab150077; Abcam) diluted in QuickBlock Secondary Antibody Dilution Buffer (P0265; Beyotime) for 2 h at room temperature. After further washes in PBS, sections were mounted on clean glass slides with glycerol and sealed with nail polish. Images were taken under Leica inverted fluorescent microscope (DMi8 automated, Leica, Germany) using 10 × objective.

### Measurement of top-down influence on contrast sensitivity of V1 neurons

Six cats were used in this experiment to measure the contrast sensitivity of V1 neurons before and after the end of c- and s-tDCS in A7 using in vivo extracellular single-unit recording techniques^17,18^.

### Recording preparation and procedure

The recording preparation was similar to that described above (see “Recording preparations” Section). After implantation of tDCS trough on the skull over A7, a small hole was drilled on the skull over V1 cortex^7,10,12^ (Horsley–Clarke coordinates: P2–P8/L0–L4) (S-Fig. 2) in the left hemisphere. After the dura was removed, recording of neuronal response in the V1 cortex was performed using a glass-coated tungsten microelectrode (with impedance of 2–3 MΩ) driven by a hydraulic micromanipulator (NARISHIGE, Japan), and the hole was covered with 4% agar. The optic discs of the two eyes were reflected onto a movable transparent tangent screen positioned 57 cm away from the eyes and overlapped with the CRT monitor for visual stimuli presentation. The central areas of both eyes were located as previously described^17,18^. V1 neurons were randomly sampled from all cortical layers in the medial bank of the lateral gyrus with the electrode penetrations within a vertical depth of 2000 µm from the pial surface. The c- and s-tDCS in A7 were applied in an interleaved order for recording of different V1 neurons. The time interval between c- and s-tDCS session was set at least 90 min to avoid overlapping of tDCS effects^24–26^.

To examine how top-down influence of A7 affects the contrast sensitivity of V1 neurons to visual stimuli, we systematically recorded the response of each neuron to its preferred moving grating stimuli (preferred orientation, motion direction, spatial frequency, temporal frequency and size) with different luminance contrast (0, 0.025, 0.05 0.1, 0.2, 0.3, 0.4, 0.5, 0.6, 0.8 and 1.0) before and after the end of c- or s-tDCS in A7. To examine if the c-tDCS induced changes in contrast sensitivity of V1 neurons could recover, we also recorded the response of V1 neurons to gradient stimulus contrast at the time point of 90 min after the end of c-tDCS when the tDCS effects vanished completely^24–26^. The grating stimuli with different contrast presented in a random order and repeated for 6 sessions (5 trials/session) both before and after the end of tDCS. Prior to the presentation of each trial of stimulus, spontaneous activity was acquired during 1 s period while the mean luminance was shown on the CRT. The recording for each studied neuron was completed within 3 min before and after the end of tDCS as well as after recovery of c-tDCS effect. At the end of recording experiment, the animals were killed by stopping its heart beat and breath through intravenous injection of pentobarbital sodium (> 100 mg kg^−1^)^24^.

### Visual stimuli and display

Moving sinusoidal grating stimuli with different luminance contrast were generated using a PC computer with the aid of Matlab programs based on Psychotoolbox extensions. All visual stimuli had a fixed mean luminance of 19 cd/m^2^ and were displayed on the CRT monitor positioned 57 cm from the animal’s eyes. The CRT monitor had a resolution of 1024 × 768 pixels and a refresh rate of 75 Hz.

### Data analysis

After the signal was amplified with a microelectrode amplifier (NIHON KOHDEN, Japan) and differential amplifier (Dagan 2400A, USA), action potentials were fed into a window discriminator with an audio monitor. The original voltage traces were digitized using an acquisition board (National Instruments, USA) controlled by IGOR (WaveMetrics, USA), and saved for online and offline analysis. The response of a cell to a grating stimulus was defined as the mean firing rate (spontaneous activity subtracted) corresponding to the time of stimulus modulation, which was used to determine the cell’s preferred orientation, motion direction, spatial frequency, temporal frequency and size^17,18^.

**Figure 7:**
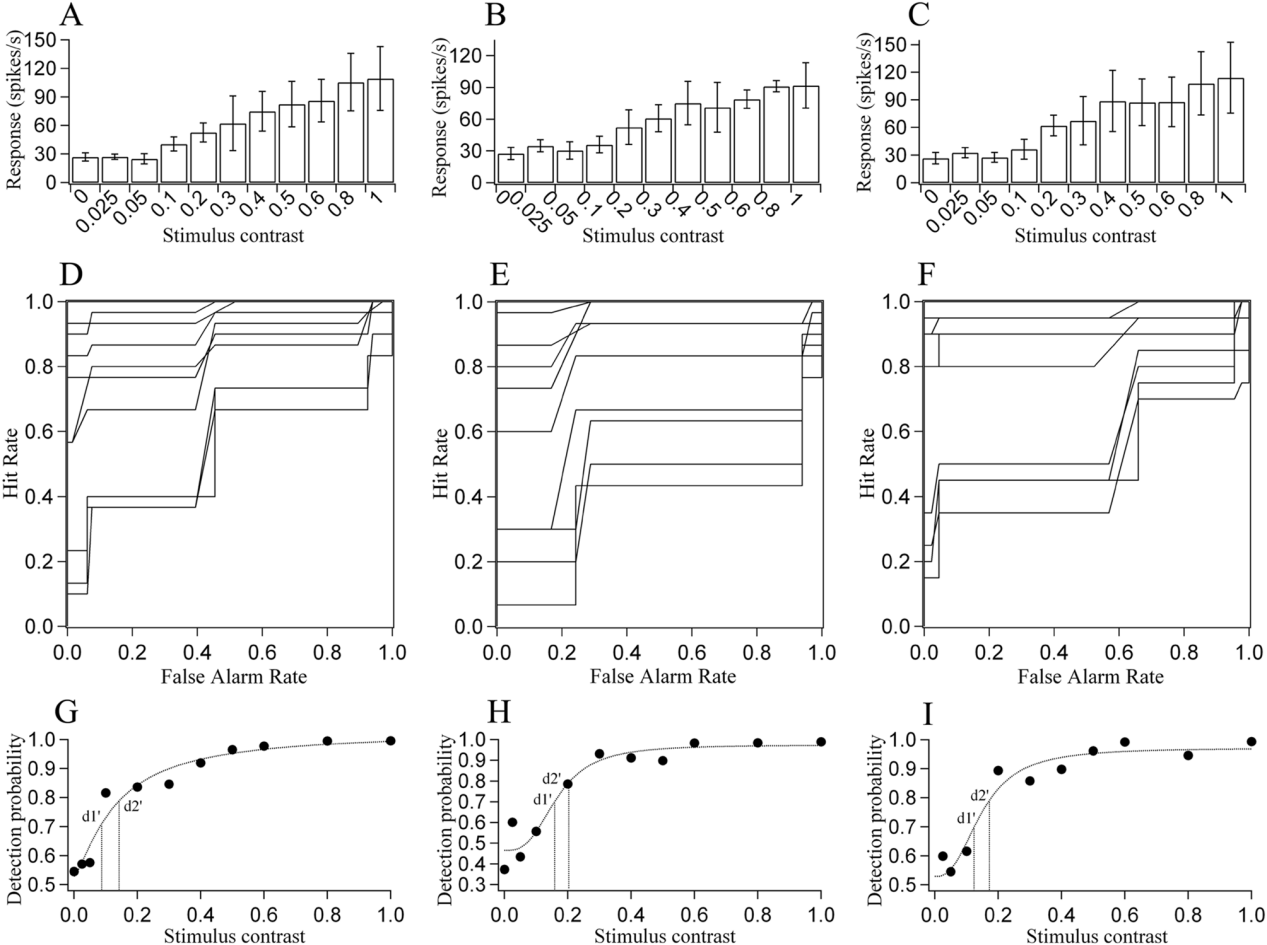
A sample cell showing the ROC analysis for measurement of the threshold contrast that the V1 neuron can respond to the visual stimuli at two performance criteria (d1′ = 70.7%, and d2′ = 79.3%) before and after c-tDCS in the area 7 (A7) of visual cortex. (**A**–**C**) Contrast response functions showing the mean neuronal response to repeated presentation (6 × 5 trials) of visual stimuli with different luminance contrast (0, 0.025, 0.05, 0.1, 0.2, 0.3, 0.4, 0.5, 0.6, 0.8 and 1.0) before (**A**) and immediately after (**B**) c-tDCS as well as after recovery from c-tDCS effect (**C**). (**D**–**F**) ROC showing the hit rate versus false alarm rate for neuronal response value distribution at different stimulus contrasts compared with that at contrast 0 before (**D**) and immediately after (**E**) c-tDCS as well as after recovery from c-tDCS effect (**F**). (**G**–**I**) showing the probability (solid circles) of the neuron in detection visual stimuli with different contrasts before (**G**) and immediately after (**H**) c-tDCS as well as after recovery from c-tDCS effect (**I**). The solid curves represent the best-fitting of detection probability versus stimulus contrast functions with Weibull equation. The dashed lines indicate the contrast threshold (TC) at two performance criterion of d1′ and d2′, respectively. Image created using Igor (version 6.3.1.2, www.wavemetrics.com).

Receiver operating characteristics (ROC) analysis^22^ was used to measure the probability that the responses of each neuron could correctly detect grating stimuli with different luminance contrast before and after tDCS as well as after recovery of tDCS affect (Fig. 7). Concisely, the neuron’s response to 30 trials (5 trials × 6 sessions) of each stimulus contrast formed a response value distribution. By scoring each of these 30 response values as “true” or “false” of being elicited by a certain stimulus contrast (0, 0.025, 0.05, 0.1, 0.2, 0.3, 0.4, 0.5, 0.6, 0.8 and 1.0) and stimulus contrast 0, the probability of the neuron in detecting a certain stimulus contrast was acquired. By fitting the detection probability versus stimulus contrast function with weibull equation, we obtained the threshold of stimulus contrast (TC) required for the neuron to detect visual stimuli at two performance criteria (d_1_′ = 70.7%, d_2_′ = 79.3%). To avoid data over- or under-estimation, neurons whose responses could not reach a TC at the performance criterion of d1′ and d2′ were excluded from data analysis. The mean TCs of neurons with different preferred SFs at two performance criteria before and after tDCS as well as after recovery of tDCS effect were compared using ANOVA and paired t-tests.

### Contrast-response function fitting

To further detect neuronal mechanisms of top-down influence, we fitted the neuronal contrast-response functions with the hyperbolic ratio equation that is widely used to characterize neuronal response to gradient luminance contrasts^17,18^:

1 $${\mathrm{R}}_{\mathrm{C}}={\mathrm{R}}_{\mathrm{max}}\times \frac{{\mathrm{C}}^{\mathrm{N}}}{\left({\mathrm{C}}^{\mathrm{N}}+{{\mathrm{C}}_{50}}^{\mathrm{N}}\right)}+\mathrm{M}$$

where Rc is the neuronal response to each stimulus contrast, M is the spontaneous activity, R_max_ is the maximum response (with spontaneous activity subtracted), C is each stimulus contrast, C_50_ is the contrast that generate half- maximum response, and N is the exponents that that control the shape of function.

Difference of best-fitting parameters, including C_50_, N, M and R_max_, before and after tDCS as well as after recovery of tDCS effect was compared using ANOVA and T-tests.

## Supplementary Information

Supplementary Figures.
