# Supplementary material for: Suppression of top-down influence decreases neuronal excitability and contrast sensitivity in the V1 cortex of cat
Source: Sci Rep. 2021 Aug 6;11:16034. doi: 10.1038/s41598-021-95407-7 (PMC8346540; doi:10.1038/s41598-021-95407-7)
Supplement: Supplementary file 1 — Supplementary Figures. [file 41598_2021_95407_MOESM1_ESM.pdf]

S-Figure 1

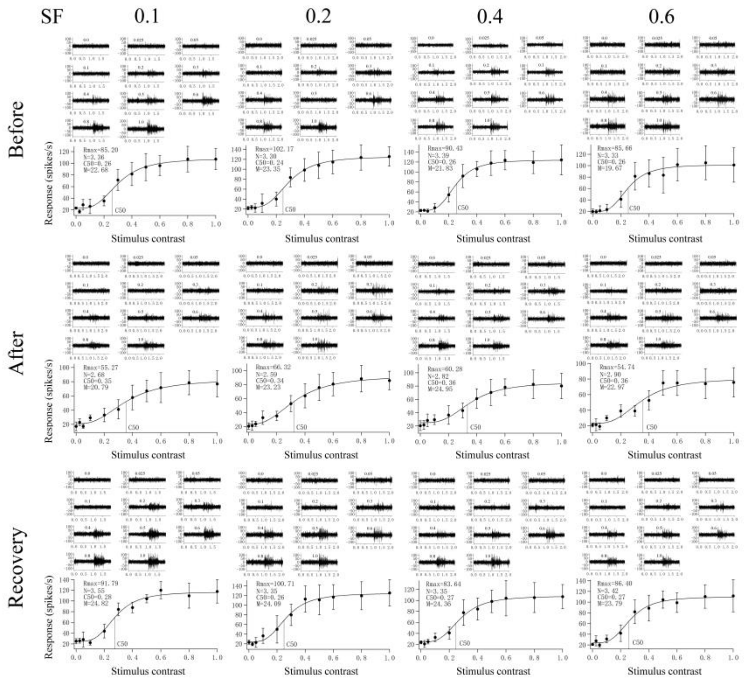

S-Figure 1. Samples of contrast-response functions and their best-fitting hyperbolic functions from V1 neurons with different preferred spatial frequencies (SF: 0.1-0.6 cycle/°) before and after c-tDCS as well as after recovery from c-tDCS effect. Voltage traces above each contrast-response functions are samples of raw data of neuronal response to grating stimuli (5 trials) of different luminance contrast (0.0-1.0) (with pre-stimulus time 1.0 s and post-stimulus time 0.5 s). The Rmax, N, C50 and M represent the best-fitting (with goodness of fit > 95%) parameter of maximum visually evoked response, exponent of contrast-response function, stimulus contrast generating the half maximum visually evoked response and spontaneous response, respectively. Image created using Igor (version 6.3.1.2, www.wavemetrics.com)。

S-Figure 2

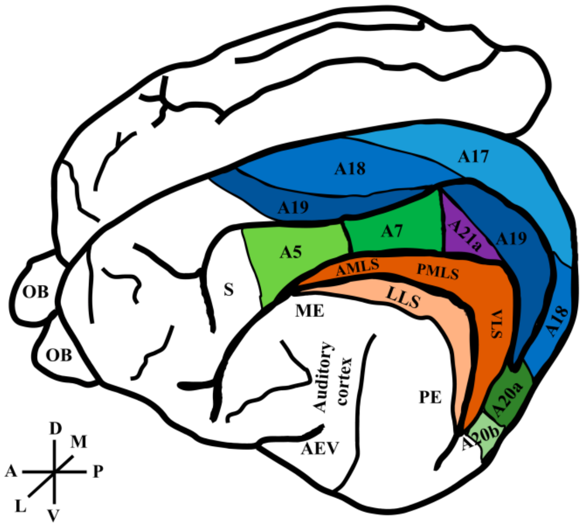

S-Figure 2. Schematic dorsolateral view of the cat brain showing locations of visual cortex of area 17 (A17: V1), area 18 (A18), area 19 (A19), area 20a (A20a), area 20b (A20b), area 21a (A21a), area 7 (A7), anteromedial lateral suprasylvian (AMLS), posteromedial lateral suprasylvian (PMLS) and ventral lateral suprasylvian (VLS) as well as nonvisual parietal cortex of area 5 (A5), somatosensory cortex (S) and auditory cortex. LLS indicates lateral lateral suprasylvian. ME and PE indicate middle and posterior ectosylvian cortex, respectively. AEV indicates anterior ectosylvian visual area (adapted from Galuske, RA, et al., 2002; Connolly, JD, et al., 2012; Bardy, C, et al., 2006). The coordinate shows the brain anterior (A)-posterior (P), dorsal (D)-ventral (V) and medial (M)-lateral (L) direction.
